# The Role of Lipoxidation in the Pathogenesis of Diabetic Retinopathy

**DOI:** 10.3389/fendo.2020.621938

**Published:** 2021-02-18

**Authors:** Josy Augustine, Evan P. Troendle, Peter Barabas, Corey A. McAleese, Thomas Friedel, Alan W. Stitt, Tim M. Curtis

**Affiliations:** ^1^ Wellcome-Wolfson Institute for Experimental Medicine, School of Medicine, Dentistry & Biomedical Science, Queen’s University of Belfast, Belfast, United Kingdom; ^2^ Department of Chemistry, King’s College London, London, United Kingdom

**Keywords:** diabetes, retina, oxidative stress, polyunsaturated fatty acids, lipid peroxidation, aldehydes, advanced lipoxidation end products, detoxification

## Abstract

Lipids can undergo modification as a result of interaction with reactive oxygen species (ROS). For example, lipid peroxidation results in the production of a wide variety of highly reactive aldehyde species which can drive a range of disease-relevant responses in cells and tissues. Such lipid aldehydes react with nucleophilic groups on macromolecules including phospholipids, nucleic acids, and proteins which, in turn, leads to the formation of reversible or irreversible adducts known as advanced lipoxidation end products (ALEs). In the setting of diabetes, lipid peroxidation and ALE formation has been implicated in the pathogenesis of macro- and microvascular complications. As the most common diabetic complication, retinopathy is one of the leading causes of vision loss and blindness worldwide. Herein, we discuss diabetic retinopathy (DR) as a disease entity and review the current knowledge and experimental data supporting a role for lipid peroxidation and ALE formation in the onset and development of this condition. Potential therapeutic approaches to prevent lipid peroxidation and lipoxidation reactions in the diabetic retina are also considered, including the use of antioxidants, lipid aldehyde scavenging agents and pharmacological and gene therapy approaches for boosting endogenous aldehyde detoxification systems. It is concluded that further research in this area could lead to new strategies to halt the progression of DR before irreversible retinal damage and sight-threatening complications occur.

## Introduction

### Diabetic Retinopathy

Diabetes mellitus (DM) is a chronic metabolic disease characterized by hyperglycemia, resulting from defects in insulin secretion, insulin action, or both. The chronic hyperglycemia of diabetes is associated with long-term damage, dysfunction, and failure of various organ systems, especially the eyes, kidneys, nerves, heart, and blood vessels (1). DM is a major healthcare issue of global epidemic proportions and is mainly divided into type 1 diabetes (T1D) and type 2 diabetes (T2D). T1D is an autoimmune disorder characterized by insufficient insulin production stemming from the loss of pancreatic β-cells (2). T2D is a metabolic disorder that is multifactorial in its etiology, resulting from insulin resistance and/or abnormal insulin secretion (3). T2D is strongly linked with changes in dietary and lifestyle factors and is increasing at an alarming rate globally (4). It is estimated that the prevalence of DM will increase substantially from the 451 million recorded cases in 2017 to 693 million by 2040 (5).

Diabetic retinopathy (DR) is the most common microvascular complication of DM and is a leading cause of blindness in the working age population worldwide (6, 7). It is a progressive sight-threatening eye disease caused by hyperglycemia, which damages the retinal microvasculature leading to vascular permeability, retinal ischemia, neovascularization, retinal detachment, and blindness (8, 9). Although hyperglycemia is central to the development of DR, it is exacerbated by hypertension (10), renal disease (11), and dyslipidemia (12). Globally, DR affects approximately one third of the general diabetic population. Within 20 years of DM diagnosis, almost all of those affected by T1D and over 60% of those affected by T2D will have at least some retinopathy (13).

Clinically, DR is classified into two progressive stages termed non-proliferative DR (NPDR) and proliferative DR (PDR). The diagnosis of DR is primarily based on symptoms or features that are visible during fundus examination. NPDR is often asymptomatic and is characterized by various pathological changes including intraretinal hemorrhages, microaneurysms, hard exudates (lipid leakage), cotton wool spots (localized disruption of axoplasmic flow), changes in vessel caliber, and ischemic spots (14). PDR involves the development of new blood vessels on the surface of the retina and is graded according to their location on the fundus and the presence or absence of vitreous hemorrhage (9).

Beyond the features visible in fundus images, NPDR is associated with a number of functional changes that usually occur within the first few years of diabetes, including alterations in rate of retinal blood flow (15), breakdown of the inner blood-retinal-barrier (iBRB) (16), and loss of neurovascular coupling mechanisms that adjust retinal capillary perfusion to neuronal metabolism (17). As NPDR progresses, vasodegenerative pathology ensues including the thickening of capillary basement membranes (BM), the loss of vascular mural cells (pericytes and vascular smooth muscle) and the formation of acellular capillaries, leading to areas of retinal non-perfusion and ischemia (18–20). Retinal ischemia induces the upregulation of pro-inflammatory cytokines and growth factors, which drive vascular permeability and pathological neovascularization (14).

Visual impairment in DR can result from PDR or the development of diabetic macular edema (DME). PDR can cause vision loss through various mechanisms including tractional retinal detachment, vitreous hemorrhaging and neovascular glaucoma (9). DME can occur at any stage of DR, where the disruption of the iBRB results in vascular leakage and the accumulation of sub- and intra-retinal fluid in the macula region of the retina, disrupting central vision (6, 13).

**Figure 1:**
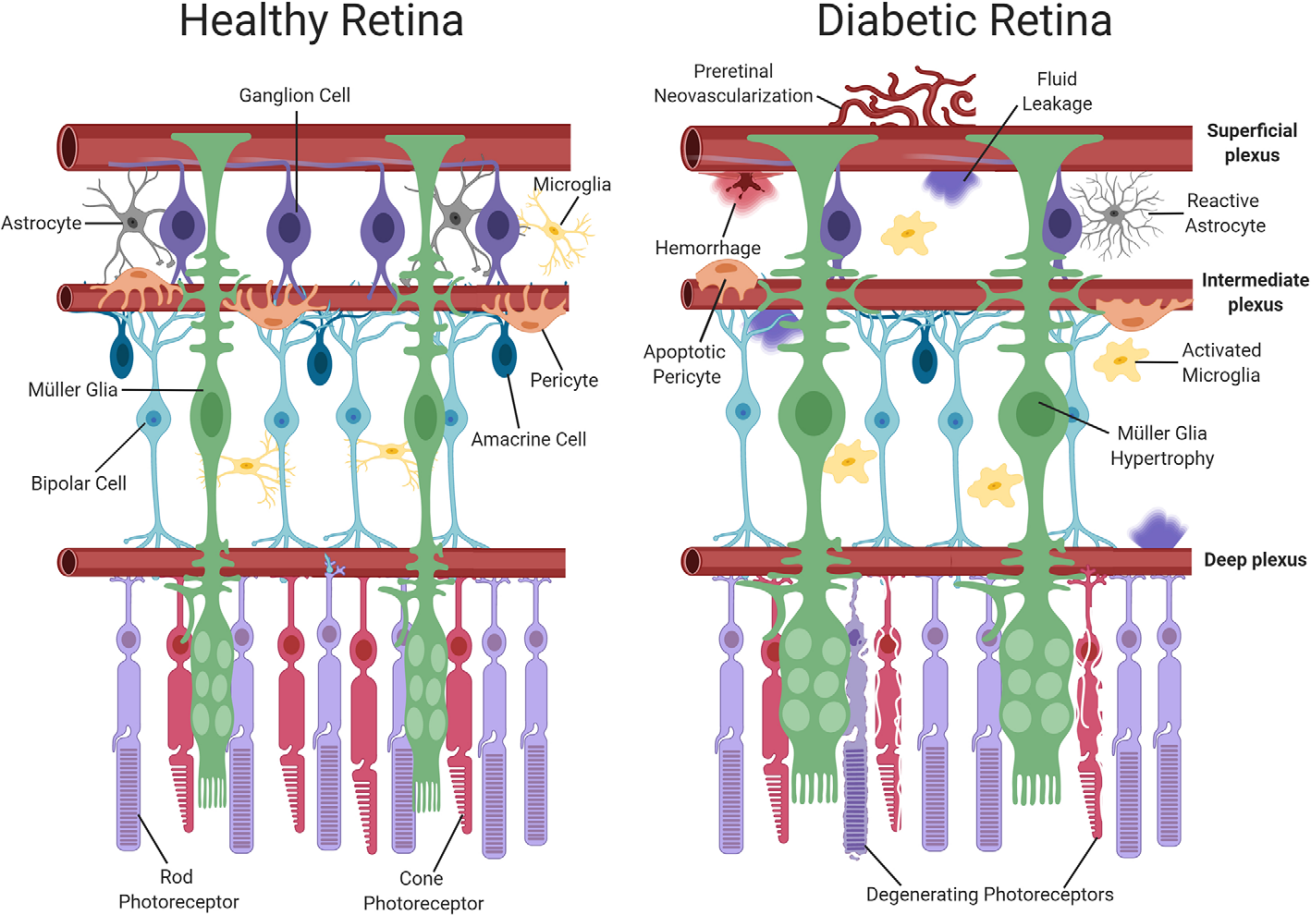
Schematic representation of neuronal and vascular structure in the healthy and diabetic retina. The healthy retina comprises of glial elements including Müller cells and astrocytes, neuronal elements including photoreceptors, ganglion, amacrine and bipolar cells, resting microglia and healthy retinal blood vessels. In contrast, the diabetic retina exhibits multiple abnormalities such as Müller cell swelling, neuronal damage, activated microglia and vascular changes (pericyte dropout, hemorrhage and neovascularization). Dysfunction of the iBRB results in the accumulation of fluid. Created with BioRender (https://app.biorender.com).

Recently, it has been suggested that DR should be re-categorized as a neurovascular rather than a microvascular complication of diabetes (21, 22). The neurovascular nature of the disease is evident even during early diabetes, with retinal neuronal dysfunction being widely reported in clinical electrophysiology studies (23–25). At a histological level, detailed analyses of post-mortem retinas from both diabetic patients and experimental animal models suggest that neurodegeneration forms an important component of DR, whereby retinal ganglion cells (RGCs), amacrine cells and even photoreceptors may be lost as diabetes progresses (26–28). Retinal glial cells also suffer during diabetes and display both functional and morphological changes (29). There are two main types of macroglial cell in the retina, namely, astrocytes and Müller glia (30). Astrocytes are largely restricted to the nerve fiber layer of the retina and play a key role in the maintenance of the iBRB and in modulating neuronal signaling (31, 32). Müller glia span the entire thickness of the retina and perform a wide range of functions including stabilization of the retinal architecture, regulation of ion homeostasis, maintenance of the iBRB, neurotransmitter recycling, and neuronal survival (33–35). Previous work in experimental animals has suggested that astrocytic intercellular communication is impaired in the diabetic retina (33), alterations which have been previously associated with the development of neuronal dysfunction (36). Müller glia display numerous changes in diabetes, with the most obvious being the induction of gliosis, characterized by cellular hyperplasia and the upregulation of the intermediate filament protein, glial fibrillary acidic protein (GFAP) (37). These cells also exhibit disturbances in their ability to regulate glutamate and K^+^ in the extracellular space and consequently have been linked to retinal excitotoxicity and the development of retinal edema during diabetes (36). As the resident immune cells of the retina, microglia also participate in the development of DR. During diabetes, retinal microglia assume a reactive phenotype, which results in the propagation of pro-inflammatory signalling and inflammatory damage (38). Because neuroretinal and glial cell alterations occur at an early stage of the disease process, these cells are thought to play an important contributory or causative role in the onset and progression of the vascular pathology associated with DR (9). **Figure 1** summarizes the main vascular, glial and neuronal changes reported in the diabetic retina.

Large, prospective, randomized clinical trials have shown that long term glycemic control reduces the risk of development and progression of DR in both T1D and T2D patients (39). Beyond the maintenance of tight glycemic control, laser photocoagulation is a late-stage treatment for PDR that sacrifices peripheral retinal regions to prevent further vision loss and preserve central vision (8). Intravitreal injection of vascular endothelial growth factor (VEGF) inhibitors is the current standard of care for patients with DME, but not all patients respond to these drugs and others become refractory to therapy (40). Aside from their destructive or invasive nature, neither treatment addresses the underlying pathophysiology of DR, with their effects being limited to preventing further late stage vasculopathy. They are also ineffective at managing or repairing the neuronal or glial cell dysfunction in early-stage DR. A greater understanding of the pathophysiological mechanisms that underlie neuroretinal dysfunction during diabetes could open up new possibilities to therapeutically manage and impede the progression of the disease.

Lipid peroxidation and lipoxidation reactions have been implicated in various neurodegenerative and cardiovascular diseases (41, 42). Lipid peroxidation refers to the oxidative degradation of lipids (43). Lipoxidation, on the other hand, describes the formation of covalent adducts between reactive products of lipid peroxidation and macromolecules such as proteins, phospholipids and DNA (44). A growing body of evidence suggests that lipid peroxidation and lipoxidation plays a key role in the development of diabetic vascular complications, including DR (45–47). In the current review, we discuss in detail the involvement of lipid peroxidation and lipoxidation reactions in the pathogenesis of DR. We also highlight therapeutic approaches that could hold promise for preventing such reactions in the diabetic retina. Further work in this area could lead to new therapeutic options for the early-stage treatment of DR, and thus negate or reduce the need for end-stage therapies such as laser photocoagulation or intravitreal anti-VEGF drugs.

## Oxidative Stress and Lipids

### Oxidative Stress

Oxidative stress results from an imbalance between the production of reactive oxygen species (ROS) and the capability of biological systems to detoxify them (48). It has long been recognized that high levels of ROS can inflict direct damage to important cellular structures including proteins, lipids, and nucleic acids (49–51). ROS can be classified into two groups of compounds called free radicals and non-radicals (52). Free radicals contain at least one unpaired electron in their outer orbit. The commonly defined free radicals include superoxide radicals $\left({\text{O}}_{2}^{•-}\right)$, hydroxyl radicals (^•^OH), hydroperoxyl radicals (HOO^•^), alkoxy radicals (RO^•^) and peroxyl radicals (ROO^•^) (53, 54). Non-radical species include hydrogen peroxide (H_2_O_2_), singlet oxygen (^1^O_2_), hypochlorous acid (HOCl), organic peroxides (ROOH) and peroxynitrite (ONOO^−^) (54, 55). ^•^OH is the most reactive and harmful ROS, being capable of accepting an electron from almost any nearby biomolecule (56, 57). The primary sources of endogenous ROS are the mitochondria, plasma membrane, endoplasmic reticulum and peroxisomes, with their generation occurring through a range of mechanisms including enzymatic reactions and the autooxidation of several compounds and xenobiotics (58). Cells deploy an antioxidant defensive system based mainly on enzymatic components, such as superoxide dismutase (SOD), catalase (CAT), glutathione peroxidase (GPx), glutathione transferase, ceruloplasmin and hemoxygenase to protect themselves from ROS-induced cellular damage (59, 60). In addition to these enzymatic antioxidants, there are several non-enzymatic antioxidants of importance, including glutathione (GSH), vitamin E and vitamin C, which can reduce ROS from a variety of sources (61). The ROS discussed above are summarized in **Table 1**.

**Table 1:** Reactive oxygen species.

Name [type] <formula>	Structure
superoxide radical [radical] $<{\text{O}}_{2}^{•-}>$	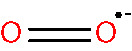
hydroxyl radical [radical] < ^•^OH >	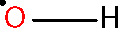
hydroperoxyl radical [radical] $<\text{H}{\text{O}}_{2}^{•}>$	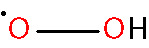
alkoxy radical [radical] < RO^• ^>	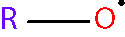
peroxyl radical [radical] $<{\text{RO}}_{2}^{•}>$	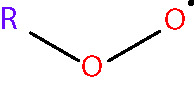
hydrogen peroxide [non-radical] < H_2_O_2_ >	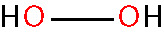
singlet oxygen [non-radical] <^ 1^O_2_ >	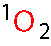
hypochlorous acid [non-radical] < HClO >	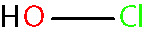
organic peroxide [non-radical] < RO_2_R^’ ^>	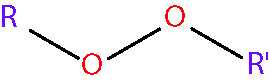
peroxynitrite [non-radical] $<\text{N}{\text{O}}_{3}^{-}>$	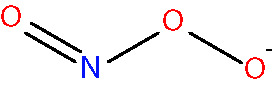

The retina displays high rates of ROS production (62, 63). Phototransduction, phagocytosis of photoreceptor outer segments by retinal pigment epithelial cells (RPE) and the oxidization of polyunsaturated fatty acids (PUFAs) in the retina leads to the chronic production of ROS (64). Oxygen consuming mitochondria in the photoreceptor inner segments also play a major role in retinal ROS production (65). During normal homeostasis, retinal cells maintain a balance between pro- and anti-oxidative signaling (66). In this regard, SOD2 plays a particularly important role by converting $O_{2}^{•-}$ into H_2_O_2_ and O_2_ in the mitochondria (67). In peroxisomes, oxidases reduce O_2_ to H_2_O_2_ during lipid breakdown, while catalases remove any excess H_2_O_2_ (66, 68). In diabetes, ROS production in the retina is significantly increased through several mechanisms including the autoxidation of glucose (69), elevated nicotinamide adenine dinucleotide phosphate (NADPH) oxidase activity (70) and mitochondrial dysfunction (71–73). The oxidative stress that results is further exacerbated by the concomitant impairment of endogenous antioxidant defenses, including SOD2 and catalase (74). Thus, during diabetes, the cells of the retina operate in a highly oxidative environment, making them especially prone to lipid peroxidation and lipoxidation reactions.

### Lipids

Lipids are a heterogeneous and ubiquitous group of compounds that form key components of all living cells. They are vital in energy metabolism, cell signaling, cell recognition and as major structural components of biological membranes (75). Lipids of biological significance can be classified into eight well-defined categories according to their distinct chemical structures. These include fatty acyls (including fatty acids), glycerolipids, glycerophospholipids, sphingolipids, saccharolipids, polyketides, sterol lipids and prenol lipids (75). Many lipid-derived products function as signaling molecules in cellular signal transduction (76). Lipid signaling can occur *via* activation of a variety of receptors, including G protein-coupled and nuclear receptors (77). The main intracellular enzymes that generate signaling lipids are lipoxygenases (LOX), cyclooxygenases (COX) and enzymes of the cytochrome P450 system (CYP).

Neural tissues are extremely rich in lipids with a 50–60% lipid content of their dry weight (78). As an element of the central nervous system, the retina displays similar characteristics (79). The lipids present in the retina play a vital role in retinal function and disease. PUFAs comprise 35 to 40% of the lipids found in the retina (80). Of these, the majority are membrane phospholipids, sphingolipids, and cholesterol (78, 81, 82). Docosahexaenoic acid (DHA; 22:6) is the most abundant PUFA in the healthy retina, with other major fatty acids such as arachidonic acid (AA; 20:4), linoleic acid (LA 18:2), oleic acid (OA; 18:1), stearic acid (SA; 18:0), and palmitic acid (PA 16:0) also present (80, 83). Listed in descending abundance, the lipid species present in the human retina include phosphatidylcholine (PC), phosphatidylethanolamine (PE), phosphatidylinositol (PI), phosphatidylserine (PS), and sphingomyelin (SM) (81). Contemporary lipidomic research is playing a key role in elucidating the precise lipid compositions (i.e. simultaneous discernment of lipid head groups and tails present) in the retina (84). DHA accounts for approximately 50% of the fatty acids in the photoreceptors, where it plays a vital role in visual transduction and protection against cell injury (85, 86). DHA is converted into neuroprotectin D1, which promotes cell survival through its anti-apoptotic and anti-inflammatory properties (87). PUFAs, particularly n-3 and n-6 species, are consistently found to be reduced in the retina during diabetes (88–90). Presently, it is unclear whether the reduction of PUFA composition in diabetes is due to increased lipid peroxidation or changes in lipid synthesis or recycling. The main lipid species present in the retina are featured in **Table 2**.

**Table 2:** Lipid species present in the retina.

Name {abbreviation} [type; subtype] <formula>	Structure
docosahexaenoic acid {DHA} [lipid tail; n‐3 polyunsaturated fatty acid] < C_22_H_31_O_2_R >	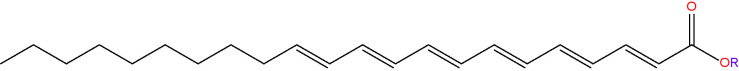
arachidonic acid {AA} [lipid tail; n-6 polyunsaturated fatty acid] < C_20_H_31_O_2_R >	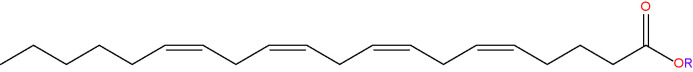
linoleic acid {LA} [lipid tail; n-6 polyunsaturated fatty acid] < C_18_H_31_O_2_R >	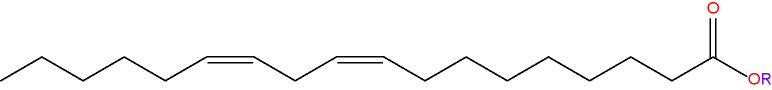
oleic acid {OA} [lipid tail; monounsaturated fatty acid] < C_18_H_33_O_2_R >	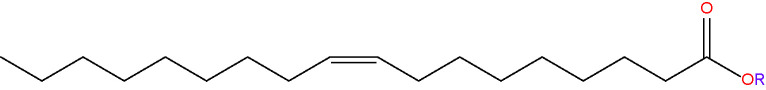
stearic acid {SA} [lipid tail; saturated fatty acid] < C_18_H_35_O_2_R >	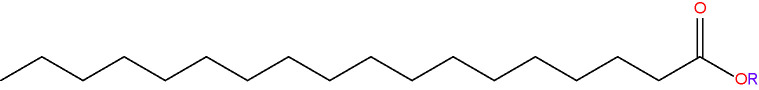
palmitic acid {PA} [lipid tail; saturated fatty acid] < C_16_H_31_O_2_R >	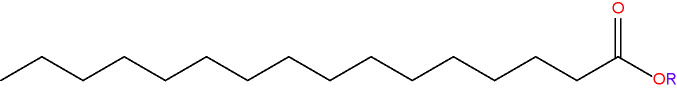
phosphatidylcholine {PC} [lipid headgroup; choline phospholipid] < RC_10_H_18_NO_8_PR' >	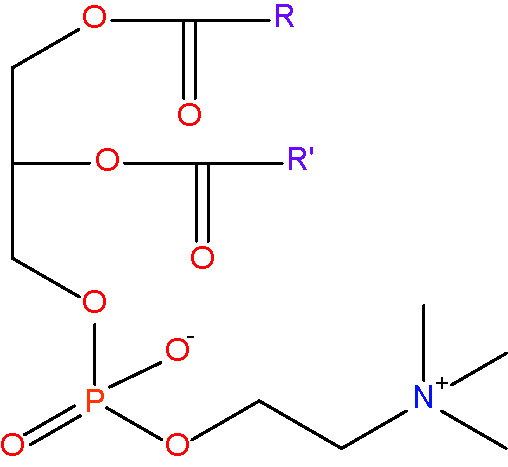
phosphatidylethanolamine {PE} [lipid headgroup; ethanolamine phospholipid] < RC_7_H_12_NO_8_PR' >	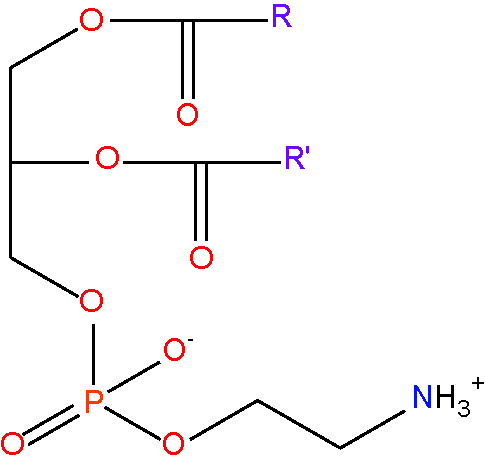
phosphatidylinositol {PI} [lipid headgroup; inositol phospholipid] < RC_11_H_16_O_13_PR'^-^ >	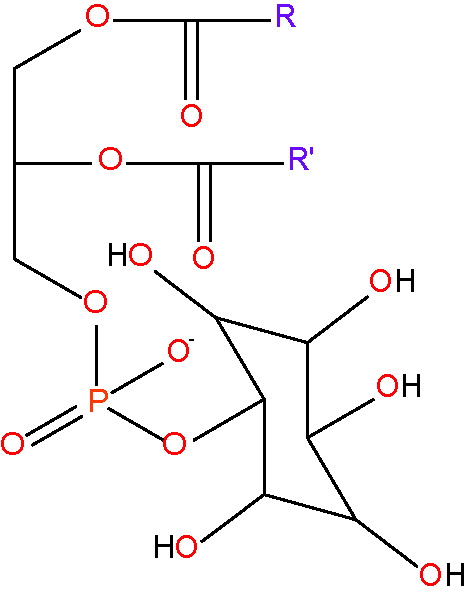
phosphatidylserine {PS} [lipid headgroup; serine phospholipid] < RC_8_H_11_NO_10_PR'^-^ >	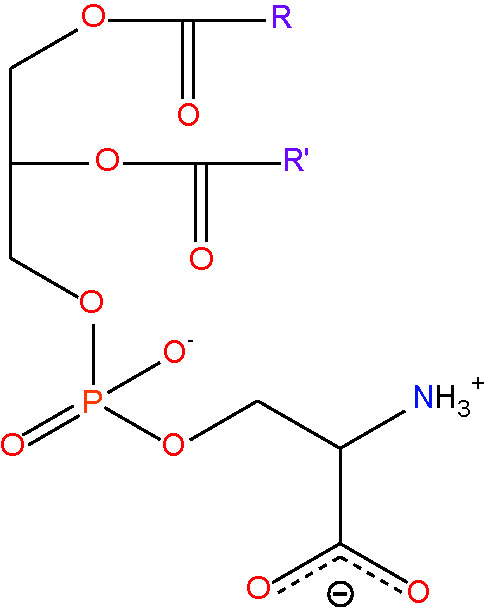
sphingomyelin {SM} [lipid headgroup + sphingosine; choline sphingolipid] < RC_24_H_48_N_2_O_6_P >	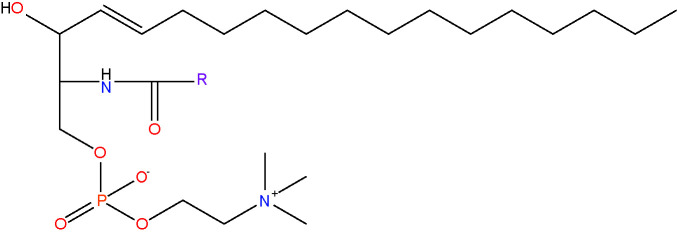

## Lipid Peroxidation and Lipoxidation

### Formation of Lipid Aldehydes and ALEs

**Figure 2:**
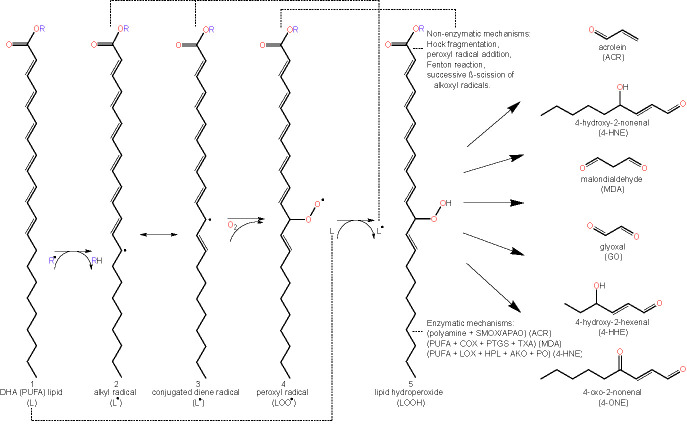
Schematic representation of lipid peroxidation and the formation of lipid aldehydes. The chemical structure of docosahexaenoic acid (DHA) is used to represent a lipid (L) undergoing peroxidation (1). Lipids (L) can become radicalized to form an alkyl radical (L^•^) (2) by some initiating radical species, (R^•^), (e.g. reactive oxygen species (ROS) *via* oxidative stress or other lipid peroxidation-derived radical molecules (L^•^, LOO^•^, etc.)) by abstracting a hydrogen from a lipid’s allylic carbon. Alkyl radicals can reversibly rearrange to form conjugated dienes (3), resulting in a higher susceptibility to oxygenation, forming a lipid peroxyl radical (LOO^•^) (4). Lipid peroxyl radicals will then accept hydrogen from any number of sources, forming lipid hydroperoxide (LOOH) (5). Abstracting another lipid’s allylic hydrogen leads to a propagation of the prior reaction in other lipids (2–5). LOOHs and PUFAs degrade to form lipid aldehydes under a plethora of mechanisms, both non-enzymatic and enzymatic. Abbreviations: polyunsaturated fatty acid (PUFA), lipid (L), lipid radical (e.g. alkyl radical/conjugated diene radical) (L^•^), lipid peroxyl radical (LOO^•^), lipid hydroperoxide (LOOH), acrolein (ACR), 4-hydroxy-2-nonenal (4-HNE), malondialdehyde (MDA), glyoxal (GO), 4-hydroxy-2-hexenal (4-HHE) 4-oxo-2-nonenal (4-ONE), spermine oxidase (SMOX), acetylpolyamine oxidase (APAO), cyclooxygenase (COX), prostacyclin synthase (PTGS), thromboxane synthase (TXA), lipoxygenase (LOX), hydroperoxide lyase (HPL), alkenal oxygenase (AKO), peroxygenase (PO).

As highlighted earlier, lipid peroxidation refers to the metabolic process whereby ROS cause the oxidative degradation of lipids (43). The process of lipid peroxidation consists of three phases called initiation, propagation, and termination (91, 92). The initiation phase involves pro-oxidants such as ^•^OH removing the allylic hydrogen and forming a carbon-centered lipid radical. PUFAs, which contain allyls (R–C=C–C–R’), are thus particularly prone to lipid peroxidation and exhibit high propagation rates for the peroxidation reaction (91). Subsequently, in the propagation regime, the lipid radical (L^•^) rapidly reacts with oxygen to form a lipid peroxyl radical (LOO^•^), which removes a hydrogen from another lipid molecule generating a new lipid radical (reviving the chain reaction) and lipid hydroperoxide (LOOH). Finally, the termination reaction may involve antioxidants such as vitamin E donating a hydrogen atom to the LOO^•^ species and forming a corresponding vitamin E radical, that reacts with another LOO^•^ forming non-radical products *via* radical disproportionation. The propagation of chain reactions continues until termination products are produced (77, 92). LOOHs are the primary products of lipid peroxidation and yield the generation of short chain unesterified aldehydes and a second class of aldehydes still esterified to the parent lipid. Steps involved in lipid peroxidation are detailed in **Figure 2**.

Although lipid aldehydes are often regarded as the final products of lipid peroxidation, it is important to appreciate that they remain highly reactive with intra- and extra-cellular macromolecules and may exacerbate oxidative stress through their reaction with membrane phospholipids. It is worth noting that lipid aldehydes are considerably more stable than ROS, and can propagate oxidative injury by diffusing to sites distant from their site of origin (93–95). These aldehydes exert a wide range of biological effects due to their ability to react with various cellular components to form relatively stable chemical adducts called advanced lipoxidation end products (ALEs) (94, 96). By forming on the nucleophilic side chains of cysteine (Cys), histidine (His) and lysine (Lys) residues, ALEs modify the structure and function of cellular proteins (94, 97). Protein adduction by ALEs has been shown to exert detrimental effects on cell function and survival by altering the activity of protein targets or by causing them to undergo rapid degradation *via* the proteosomal pathway (98). **Table 3** shows the main lipid aldehydes and ALEs formed during lipid peroxidation and lipoxidation reactions.

**Table 3:** Lipid aldehydes and ALEs.

Lipid aldehyde {abbreviation} [type]	Advanced lipoxidation end-products (ALEs) {abbreviation}
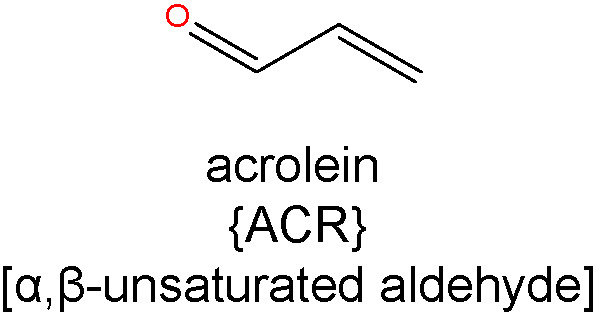	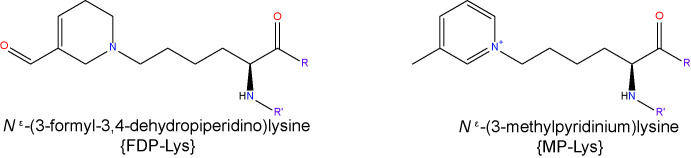
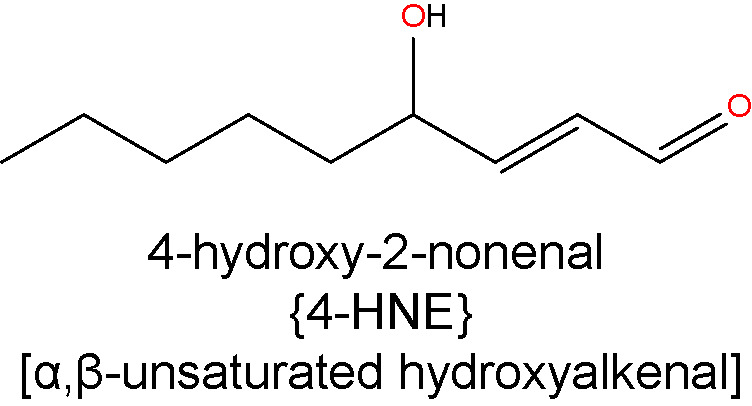	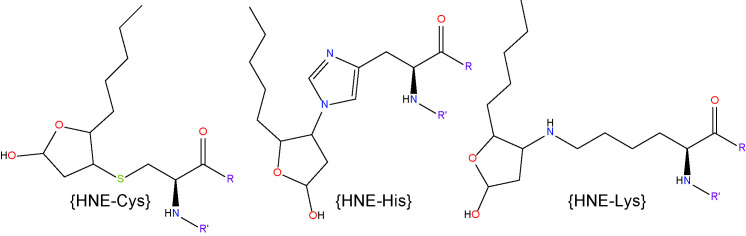
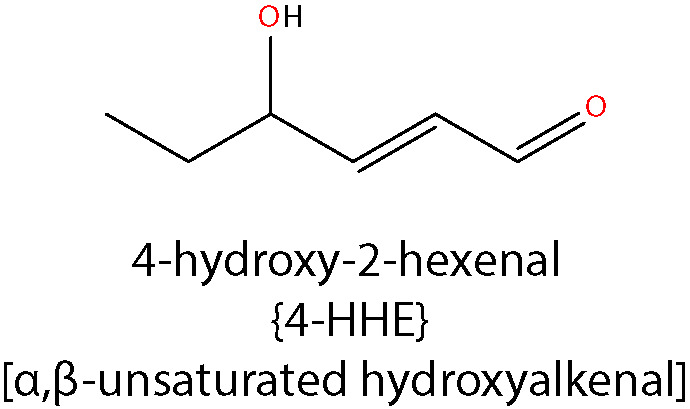	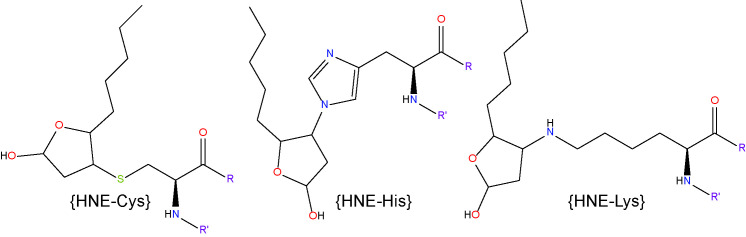
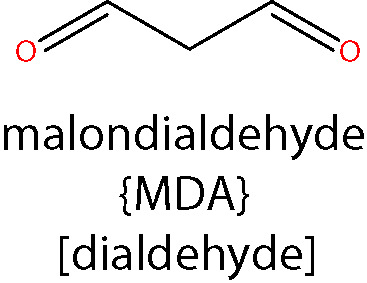	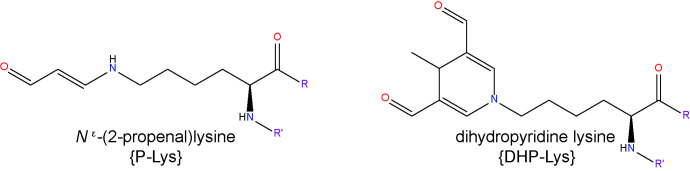
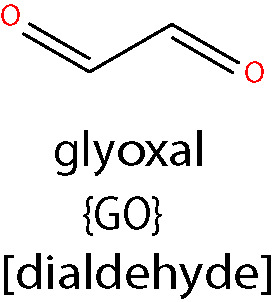	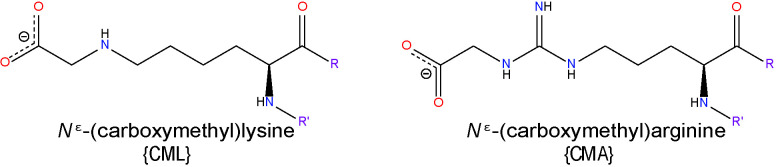
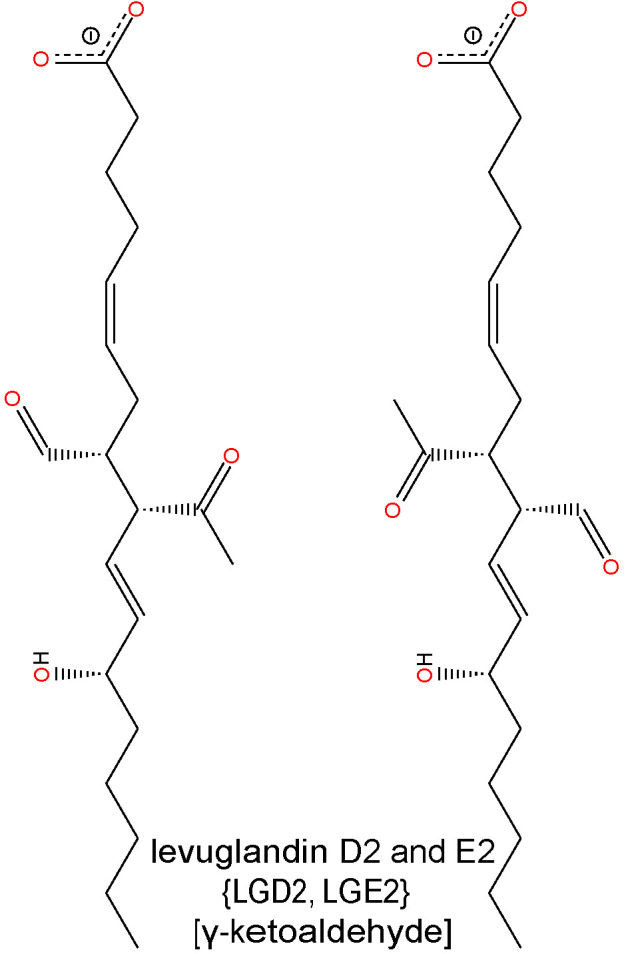	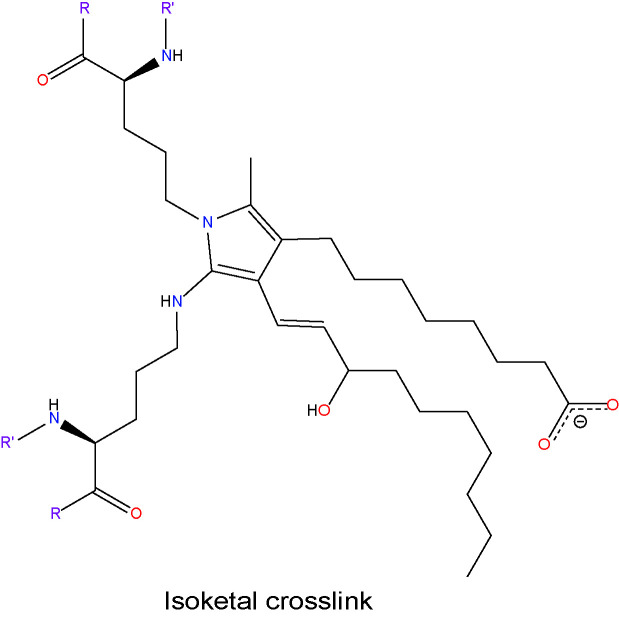

While ROS and reactive aldehydes have been widely implicated in the development and propagation of retinal diseases, other lines of research have studied their role in normal immunological processes and physiological cell signaling. It is well known, for example, that neutrophils and macrophages use high concentrations of ROS and reactive aldehydes (micromolar or greater) as part of their arsenal to destroy bacteria and other microorganisms (99–103). Under physiological conditions, these molecules are normally present at sub-micromolar concentrations in most cells and tissues and serve as important signaling agents, activating antioxidative-, endoplasmic reticulum (ER) stress-, autophagic- and pro-apoptotic pathways (104, 105). Consequently, they play a critical role in adjusting the cell’s redox state, metabolism and survival. ROS seem to directly switch off phosphatases PTP1b, PTEN and MAPK (106); thus a change in the level of ROS concentration could shift the balance of tyrosine phosphorylation-dependent signals. ROS and reactive aldehydes may also fulfil additional roles in regulating gene expression and cell-to-cell communication (107, 108), the latter being particularly relevant in the case of the more stable, longer-lived lipoxidation products and their adducts. While there is little doubt that the presence of high concentrations of ROS, reactive aldehydes and ALEs is detrimental, there remains insufficient information on the consequences of reducing their levels below those that normally occur physiologically. Indeed, a lack of insight into the beneficial effects of these molecules has been cited as the probable cause of unfavorable outcomes in some human clinical trials using antioxidant therapies (109–111).

### Measurement of Lipoxidation

Lipid peroxidation products can be detected in their free form and the most common techniques used include spectrophotometric methods and gas liquid chromatography (GLC) or high-performance liquid chromatography (HPLC) coupled to mass spectrometry (MS) (112). However, these products are more likely to be present in biological samples in the form of ALEs (97). The common methods for detection and quantification of protein-bound ALE adducts are proteomic methods based on MS and antibody-dependent techniques (113). Currently, MS is the method of choice because it is possible to detect the mass shift caused by adduction and acquire fragmentation spectra that can be used for the identification of specific fragment ions for each modified amino acid within the protein sequence. Antibody-dependent techniques such as immunohistochemistry, enzyme-linked immunosorbent assay (ELISA), and gel-based approaches are time consuming and site-specific characterization of oxidative modifications is often not possible using these methods (96, 114). The thiobarbituric acid (TBA) reactive substances (TBARS) assay is the most frequently used technique to measure the concentration of malondialdehyde (MDA), where TBA reacts with MDA to form a colored chromogen fluorescent red adduct which is quantified using spectrophotometry. However, there have been concerns about the specificity and validity of this assay, as TBA may react with many other compounds in addition to MDA, generating further oxidation and inaccurate estimation of quantitative results (115).

## Lipid Peroxidation, ALEs and DR

In recent years, strides made in the research of lipid peroxidation have shed light on the role of this process in the pathogenesis of ocular diseases. Whilst most work to date has focused on DR, there is also evidence supporting a possible role for lipid peroxidation and ALE formation in the development of other major eye diseases, including age-related macular degeneration (AMD), cataract formation, glaucoma, retinopathy of prematurity, retinitis pigmentosa and uveitis (47). A body of evidence from our group and others strongly suggests that lipid aldehydes and ALEs are key contributors to the development of DR (47, 116). Correlations between lipid peroxidation products detected in the blood and retinal changes have been reported in the streptozotocin (STZ)-induced rat model of experimental T1D (45, 117). Higher LOOH levels have also been found in retinas from human subjects with diabetes and STZ-diabetic rats (117, 118). This work has been followed up by several studies investigating how levels of specific lipid peroxidation products, primarily the lipid aldehydes, acrolein (ACR), 4-hydroxy-2-nonenal (4-HNE), MDA and glyoxal (GO), may correlate with disease progression. In the next section, we discuss the experimental and clinical evidence supporting a role of lipid aldehydes (α, β-unsaturated aldehydes, dialdehydes and γ-ketoaldehydes) and their ALEs in the initiation and progression of DR.

### α, β-Unsaturated Aldehydes

#### Acrolein

ACR is the strongest electrophile of the α, β-unsaturated aldehydes and exhibits the highest toxicity and reactivity with protein nucleophiles (119). ACR is produced by both cellular biochemical processes and oxidative reactions outside of the body. The source of endogenous ACR is not only peroxidation of lipids, but also metabolism of amino acids, polyamines, and drugs (41, 119, 120). Exogenous ACR is a ubiquitous environmental pollutant, originating from the incomplete combustion of coal, petrol, plastics, wood and tobacco (120). Studies by Uchida et al. showed evidence that ACR is a product of lipid peroxidation reactions, from studies exploring the metal-catalyzed (Cu^2+^) oxidation of LDL and autoxidation of PUFAs (41). Under conditions of oxidative stress and inflammation, ACR is produced endogenously by myeloperoxidase-mediated degradation of threonine and the amine oxidase-mediated degradation of spermine and spermidine (120). Phagocytic white blood cells eliminate invading pathogens such as viruses and bacteria by utilizing the myeloperoxidase system, which catalyzes the formation of HOCl, from chloride and H_2_O_2_. HOCl reacts with threonine to generate ACR (via 2-hydroxypropanal) and this mechanism of ACR generation is thought to mediate permanent tissue damage at sites of inflammation (121). ACR production *via* the polyamine pathway arises due to the activity of two enzymes, namely, spermine oxidase (SMOX) and acetylpolyamine oxidase (APAO) (122, 123). SMOX oxidizes spermine to produce H_2_O_2_, spermidine, and 3-aminopropanal (3-AP), with the latter breaking down to form ACR. APAO generates ACR through the formation of 3-acetamidopropanal (3-AAP), which together with H_2_O_2_, results from its metabolism of N1-acetylspermine or N1-acetylspermidine to spermidine or putrescine.

The major adduct formed upon reaction of ACR with protein has been identified as the novel Lys product, *N* ^ϵ^-(3-formyl-3,4-dehydropiperidino)lysine (FDP-Lys), which requires the attachment of two ACR molecules to one Lys side chain (41). ACR also forms ALEs with His and Cys, as detected in oxidized low-density lipoproteins (41). Unlike most other ALEs, FDP-Lys is not a stable end product, but a reactive intermediate that covalently binds to thiols, thereby initiating or exacerbating oxidative stress through the depletion of the endogenous antioxidant GSH (124). More recently, an ACR-Lys adduct called *N* ^ϵ^-(3-methylpyridinium)lysine (MP-Lys) has been identified as a major antigenic adduct generated in reactions of ACR with proteins. In contrast to FDP-Lys, the MP-Lys adduct is a highly stable end-product and does not readily react with cellular nucleophiles (125).

Due to the high reactivity of ACR and availability of reactants, it is generally not regarded as a useful biomarker in its free form. To circumvent this issue, the accumulation of ACR-derived ALEs is frequently used as an alternative approach, providing a time-integrated measurement of lipid peroxidation (41). Studies from our group compared serum and hemoglobin levels of the ACR adduct, FDP-Lys, in T1D and T2D patients with or without DR pathology (126). Our data showed that while the serum levels of FDP-Lys did not correlate significantly with the presence of DR, hemoglobin bound FDP-Lys was positively associated with the stage and severity of the disease. Given the longer lifespan of erythrocytes in comparison to the half-life of serum albumin (~120 days versus ~20 days respectively) the disparity between our serum and hemoglobin data is most likely explained by a more efficient removal of adducts from dissolved blood protein components.

**Figure 3:**
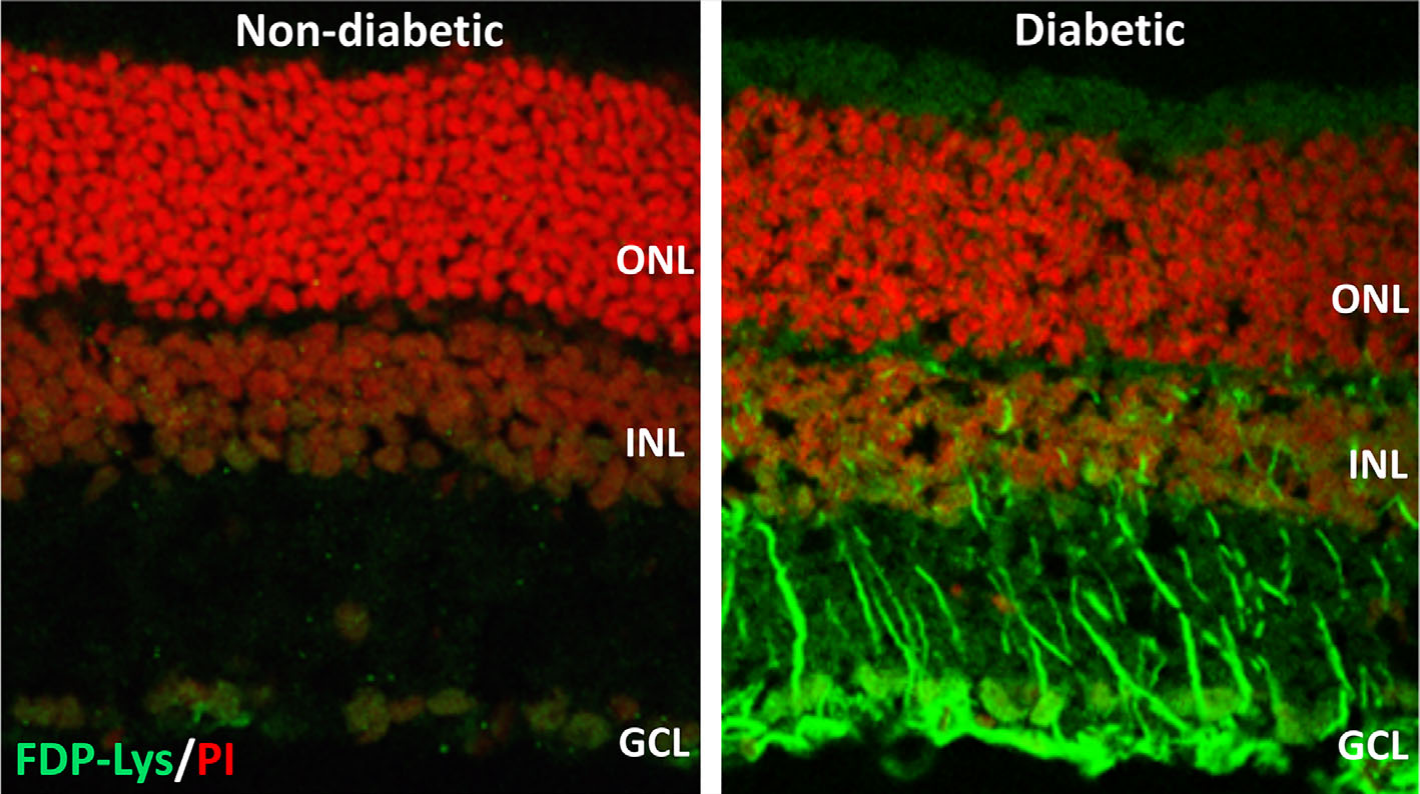
Representative confocal images showing FDP-Lys immunoreactivity (green) in transverse retinal cryosections from a non-diabetic and STZ-diabetic rat of 4-months disease duration. Nuclei were counterstained with propidium iodide nuclear stain (red). In the diabetic animal, a marked accumulation of FDP-Lys was observed in the Müller glia radial fibers and neurons of the inner retina. ONL, Outer Nuclear Layer; INL, Inner Nuclear Layer; GCL, Ganglion Cell Layer.

**Figure 4:**
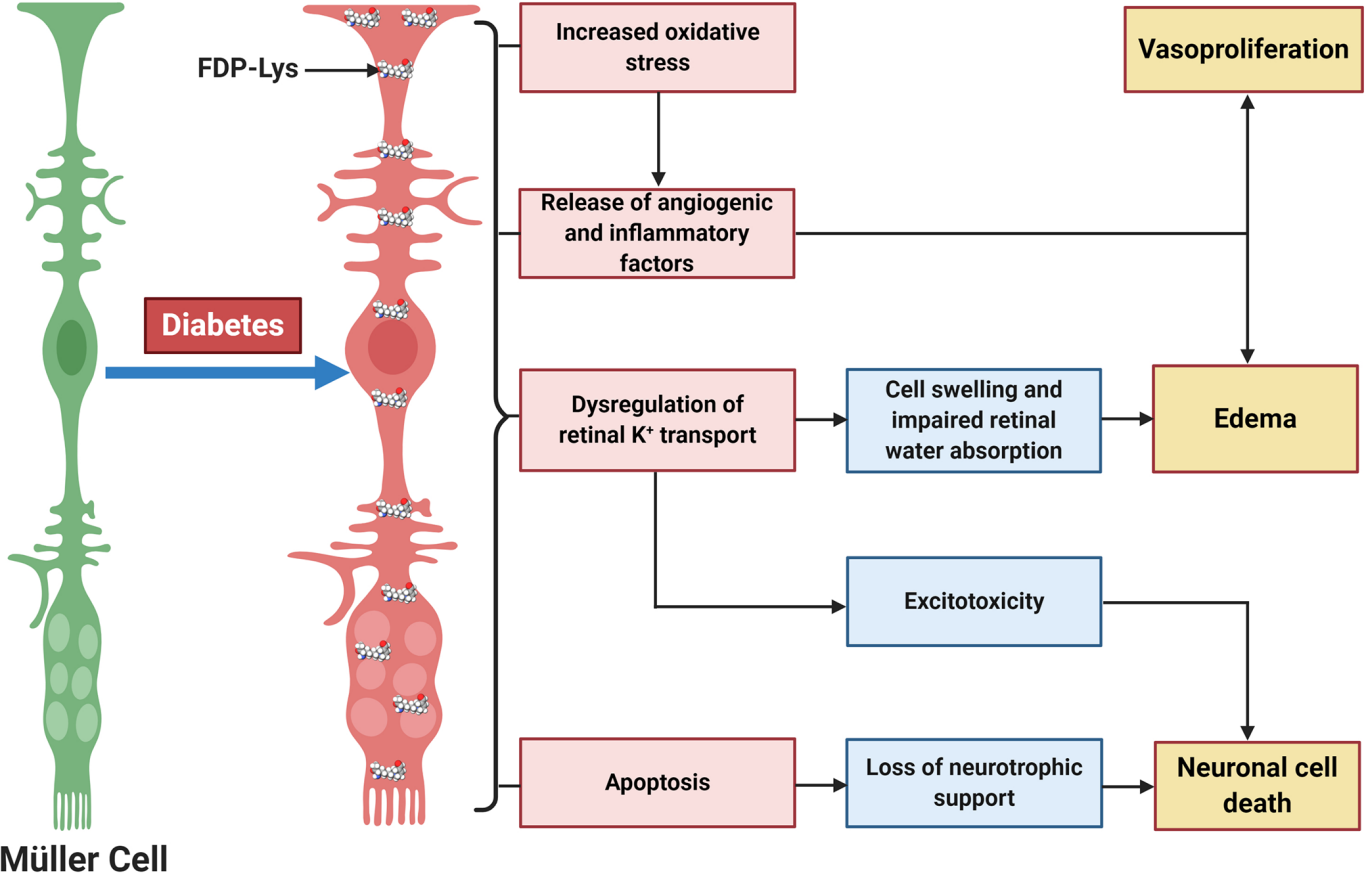
Schematic diagram showing the central role of Müller glia FDP-Lys accumulation in the pathogenesis of DR. FDP-Lys accumulation in Müller glia induces increased oxidative stress, the release of angiogenic and inflammatory factors, dysregulation of retinal K^+^ transport, and apoptosis in the diabetic retina which contributes to the sight-threatening complications of DR. Created with BioRender (https://app.biorender.com).

We went on to investigate whether ACR-protein adducts contribute to the pathogenesis of DR using the STZ-diabetic rat model (127). Specifically, we began by examining the retinal distribution and accumulation of FDP-Lys after 4 months of experimental diabetes. FDP-Lys immunoreactivity increased significantly in diabetic animals whilst being only sparsely present in healthy, control retinas (127). During diabetes, FDP-Lys accumulated predominantly within the Müller glial cells and neurons of the RGC and inner nuclear layers (**Figure 3**). Investigation of multiple, monthly time-points allowed for the determination of the spatiotemporal pattern in which FDP-Lys accumulates, initially occurring at the inner limiting membrane with a gradual spread distally to Müller cell radial processes as the disease progresses. *In vitro* cultures of MIO-M1 human Müller cells demonstrated how exposure to FDP-Lys induced increased oxidative stress, impacted K^+^ ion homeostasis and caused upregulation of VEGF and proinflammatory cytokines (102, 127). As discussed earlier (*Diabetic Retinopathy*), these *in vitro* features of Müller cell dysfunction concur with the pathology seen in these cells during DR (20). A schematic diagram showing how Müller cell FDP-Lys accumulation contributes to the pathogenesis of DR is shown in **Figure 4**.

Our experimental work into the role of FDP-Lys in DR has been supported by studies from other groups. FDP-Lys was reportedly elevated within the vitreous of human PDR patients in comparison to those of non-diabetic individuals, and immunohistochemical analysis of retinal fibrovascular tissue demonstrated co-localization of FDP-Lys with Müller cell, endothelial and pericyte markers (128, 129). Furthermore, the same group showed that *in vitro* exposure of human endothelial cells to ACR increased oxidative stress and decreased cell viability, similar to what we observed in our Müller cell FDP-Lys investigations.

As mentioned above, ACR and its associated adducts may be produced *via* the polyamine pathway (130). The polyamine spermine, a precursor of ACR in the polyamine catabolic pathway, was reported to reach a 15 times increased concentration in the vitreous of PDR patients (131). Furthermore, a very recent study showed that pharmacological inhibition of SMOX significantly reduced retinal FDP-Lys accumulation, neurophysiological dysfunction and neurodegeneration in a mouse model of diabetes (132). These findings indicate that FDP-Lys accumulation in the diabetic retina may be related not only to lipoxidation, but also to dysregulation of the polyamine pathway. Thus, overall, it seems that accumulation of ACR and its FDP-Lys adduct may have an important role in DR and both of these molecules may represent potential therapeutic targets for the early-stage treatment of this disease.

#### 4-Hydroxy-2-Nonenal

4-HNE represents the most abundant α, β−unsaturated hydroxyalkenal (i.e. a hydroxylated unsaturated aliphatic aldehyde) formed among the products of lipid peroxidation reactions (98). It is produced *via* enzymatic and non-enzymatic oxidation of n-6 PUFAs, including AA and LA (77). 4-HNE is considered one of the most toxic products of lipid peroxidation due to its rapid reaction with proteins and other macromolecules (133). However, due to cellular, high-capacity enzymatic detoxification processes, 4-HNE is largely and rapidly metabolized and only a small fraction of it escapes to react with biomolecules (134). 4-HNE reacts mainly with sulfhydryl groups of thiols in proteins to form Michael adducts and these adducts subsequently undergo secondary reaction to form cyclic hemiacetals (119, 135). It also reacts with primary amines and phosphatidylethanolamine to form Michael and pyrrole adducts (136, 137), and with DNA bases to form exocyclic DNA adducts (138).

In terms of glycemic control, recent work has shown that low, non-toxic concentrations of 4-HNE can activate peroxisome proliferator-activated receptor δ (PPARδ) complexes and thereby increase the release of insulin from cultured β pancreatic cells (139). However, when 4-HNE levels rise in response to nutrient overload or obesity, this may contribute to the onset and progression of insulin resistance and β cell dysfunction (140). Within the context of DR, clinical studies have reported elevated levels 4-HNE in the blood of diabetic patients with retinopathy compared to those without retinopathy and healthy controls (46). There is also evidence showing that 4-HNE and 4-HNE-derived ALEs increase in the retinas of rats rendered diabetic for 4-6 weeks (118, 141). Another study confirmed these findings and showed that 4-HNE may contribute to the pathogenesis of DR by activating the WNT signaling pathway through stabilization of the WNT co-receptor LRP6 (142). Other studies have linked 4-HNE to retinal hemodynamics changes during DR. Retinal perfusion deficits during early diabetes are thought to be mediated, at least in part, through the reduced activity of large-conductance Ca^2+^-activated K^+^ (BK) channels on the retinal vascular smooth muscle cells, causing vasoconstriction (131, 143). 4-HNE impairs BK channel function in rat retinal arterioles, as demonstrated by reduced vasodilatory responses to the BK channel opener, BMS-191011 (132). 4-HNE accumulation may also be involved in the development of DME. Evidence for this comes from studies in cultured retinal Müller glia, where it has been shown that 4-HNE triggers downregulation of Kir4.1 and AQP4 channels (144, 145), two of the main proteins associated with Müller glia swelling and the disruption of water homeostasis in the diabetic retina (146, 147). 4-HNE exposure can also result in endoplasmic reticulum stress, mitochondrial dysfunction and apoptosis in cultures of human retinal capillary pericytes and Müller glia (148, 149). Our own immunohistochemical analysis of 4-HNE-His levels in rat retinas after 4 months of experimental diabetes found no evidence for accumulation of this adduct (127). The reasons for this are presently unclear, although the duration of diabetes was longer in our experiments compared to those conducted by others. Whether the retina adapts, enabling it to cope better with excess 4-HNE production during longer-term diabetes, warrants future investigation.

### Dialdehydes

#### Malondialdehyde (MDA)

MDA is a moderately toxic dialdehyde molecule (LD_50_ 600-650 mg/kg in rats (150), and due to its relative stability compared to the aldehydes mentioned above, it is usually considered as a good biomarker of oxidative stress and lipid peroxidation (151). MDA originates from lipid peroxidation of PUFAs (152) containing at least two C=C double bonds flanking a single C-C link (i.e. a conjugated diene) such as AA or DHA (153). The reactivity of MDA is pH-dependent and at physiological pH, it rapidly forms enolates, which are of lower reactivity and do not react as avidly with nucleophilic species as other aldehydes (119). However, MDA exists as β-hydroxyacrolein at lower pHs, exhibiting a higher reactivity, readily reacting with Lys residues of proteins to form the enaminal type MDA adduct, *N* ^ϵ^-(2-propenal)lysine, and the fluorescent product, dihydropyridine (DHP) lysine (154, 155).

Being one of the most commonly used biomarkers of lipid peroxidation, there is a significantly larger number of reports on the assessment of MDA levels in DR when compared to 4-HNE or ACR (156). An elevation of MDA concentration was found in the blood of DR patients by several studies (46, 157–159). Moreover, there is evidence demonstrating correlations between serum MDA levels and the severity of DR, further substantiating a possible role for MDA in its pathogenesis (160, 161). By normalizing MDA levels to SOD as a proxy to obtain a ratio of lipid peroxidation stress to cellular antioxidant capacity, diabetic individuals with DR were found to have significantly reduced SOD/MDA ratios when compared to those without this complication (162). There is some evidence pointing to an increase of MDA levels in human eyes in diabetic patients. A high-performance liquid chromatography (HPLC)-based analysis of sub-retinal fluid collected from subjects affected by retinal detachment, found increased MDA concentrations in those also affected by diabetes (163). MDA levels were also reportedly increased in the lenses of diabetic individuals undergoing cataract surgery (164).

Some recent studies, however, have cast doubt on the involvement of MDA in the development of DR. A study of the aqueous humor and serum of T2D patients, found that neither were significantly different from controls (165) and investigation of MDA levels in both the vitreous humor and serum of PDR patients, found increased concentrations only in the vitreous, but not in the serum (166). In STZ-diabetic rats, we were unable to detect an elevation of MDA adducts in retinal sections using confocal immunolabelling techniques (127). Further work is clearly necessary to substantiate the notion that MDA contributes to the pathogenesis of DR and thus may be useful as a biomarker for the development of this condition.

#### Glyoxal

GO, generated by lipid peroxidation of AA and LA (98), is the simplest dialdehyde (167). As an aldehyde, it is unstable, readily forming hydrates and polymers in solution. As such, it is a natural alkylating fixative, forming crosslinks between biomolecules. GO reacts with Lys residues of proteins to form *N* ^ϵ^-(carboxymethyl)lysine (CML) and Arg residues to give rise to dihydroxyimidazolidine which is slowly degraded to *N* ^ω^-carboxymethyl-arginine (CMA) (168). Since GO can also be produced during glycation reactions, adducts formed by this aldehyde are often termed either advanced glycation end products (AGEs) or ALEs (169). The GO-derived CML adduct has been the most widely studied within the context of DR. Various clinical studies have focused on the quantification of CML in patient serum and tissues using ELISA-based methods and have demonstrated that levels of CML in serum and aqueous humor correlate with the onset or grade of DR (170, 171). CML has also been extensively quantified in the retinas of post-mortem human donor eyes and those from experimental rodent models of diabetes. These studies have revealed that diabetes significantly enhances the accumulation of CML-modified proteins throughout both the vascular and neural tissue components of the retina, causing impairment of normal retinal function and cell survival (116, 172).

### Other Aldehydes

Most studies to date have focused on the production, accumulation, and effects of ACR, 4-HNE, MDA, and GO in DR. However, there are several other aldehydes produced by lipid peroxidation, such as 4-hydroxy-2-hexenal (4-HHE), 4-oxo-2-nonenal (4-ONE), and isoketals (IsoKs—e.g. levuglandin D2 (LGD2) and levuglandin E2 (LGE2)), the roles of which have yet to be determined in DR. 4-HHE is structurally similar to 4-HNE, and forms stable ALE adducts with His, Cys or Lys residues of proteins (173). 4-HHE is generated through the peroxidation of n-3 fatty acids and it has been reported to induce both cell dysfunction and cell death *in vitro* (174). 4-HHE is considered less toxic than 4-HNE due to its lower lipophilicity and reduced chemical reactivity (47, 175). 4-ONE is formed by the peroxidation of LA, but it is also produced by the two-electron oxidation of 4-HNE (176). It is a potent crosslinker of proteins and nucleotides and does so an order of magnitude more rapidly than 4-HNE. Finally, IsoKs are formed by the peroxidation of AA and rearrangement of endoperoxide intermediates of the isoprostane pathway (177, 178). They are usually found as protein adducts, as the free form is highly reactive with amine groups. Thus, there is still a considerable amount of work to be done in determining the involvement of other lipid aldehyde species in the pathogenesis of DR.

## Endogenous Detoxification of Lipid Aldehydes and DR

Cells have evolved a range of protective mechanisms that enable the rapid metabolism and detoxification of lipid aldehydes produced during lipid peroxidation reactions. In most cells, lipid aldehydes are oxidized to acids, reduced to alcohols or conjugated with cellular nucleophiles such as ascorbic acid, glutathione and carnosine (179).

The oxidation of lipid aldehydes to their corresponding acids is primarily catalyzed by enzymes of the aldehyde dehydrogenase superfamily (ALDH). The ALDH superfamily comprises a wide variety of NAD(P+) dependent enzymes that irreversibly catalyze the oxidation of aldehydes to their respective carboxylic acids (180). Humans express 19 isoforms of ALDHs, which are grouped into several classes (ALDH 1–9, 16 and 18). ALDH 1, 2 and 3 family members play a central role in oxidizing lipid peroxidation products (95, 181). Members of the ALDH superfamily are expressed in a species and tissue specific fashion. ALDHs also play a vital role in the modulation of cell proliferation, differentiation, and survival, especially through participation in retinoic acid synthesis (182). There remains a significant amount of work to be done in assessing the substrate specificity of many of these enzymes. A large proportion of them, however, seem to have multiple roles in aldehyde processing with varying catalytic efficiency for the conversion of lipid-derived electrophiles (181).

The enzymes responsible for reducing lipid aldehydes to alcohols include the aldo-keto reductase (AKR) superfamily, alcohol dehydrogenase family and the short-chain dehydrogenase/reductase family (179). Conjugation reactions such as glucuronidation and sulfation, subsequently prevent re-oxidation of the alcohol back to its parent aldehyde (181). AKRs comprise a large family of enzymes, which are catalytically active in the modification of a range of substrates. These enzymes are NAPDH-dependent oxidoreductases which catalyze the reduction of a wide range of aldehydes and ketones to primary and secondary alcohols, respectively. There are 13 isoforms that are known to be expressed in humans and several of those belonging to families 1 and 7 are associated with the reduction of aldehydes (183). AKR1B1, or aldose reductase, is perhaps best known for its pathological role in diabetic vascular complications by converting excess glucose to its sugar alcohol, sorbitol (184). Paradoxically, however, AKR1B1 is a relatively poor catalyst for glucose reduction and in recent years it has become increasingly recognized that its normal cellular function is linked with aldehyde metabolism (184, 185). AKR1a1, AKR7A3, AKR7a2, AKR1B1 and AKR1B10 have been identified as ACR-metabolizing enzymes (186–189). Other enzymes of the AKR family for which aldehyde reducing capabilities have been established include AKR1C1 and its rat homolog AKR1C9, which have been shown to efficiently catalyze the oxidation of 4-HNE *in vitro* (190). In addition, AKR7A1 and AKR7A2 when overexpressed in V79 cells (Chinese hamster lung fibroblasts) have been reported to protect against the harmful effects of ACR (186). AKR enzymes display very broad substrate specificities, although the vast majority have been shown to have a role in aldehyde detoxification (191).

We have recently screened for the mRNA expression of ALDH and AKR enzymes in the retina (95). Of the enzymes examined, most ALDH family members were identified including ALDH1a1, ALDH2, ALDH3a1, ALDH3a2, ALDH9a1, ALDH18a1, and ALDH1L1. Fewer AKR enzymes were apparent, although AKR1b1, AKR1c19, and AKR7a2 could be detected. Interestingly, of the various ALDH and AKR enzymes that we have studied in the retina to date, most appear to be predominantly localized to the Müller glia, including ALDH1a1, ALDH2 and AKR1b1 (95). These findings are consistent with the notion that a key, previously unrecognized, function of these cells, is to protect the retina through the detoxification of lipid peroxidation products. During diabetes, we observed a general trend towards downregulation of ALDH and AKR gene expression in the retina, including ALDH1a1, which was also markedly reduced at the protein level in Müller cells. ALDH enzymatic activity was also substantially lower in whole retinal lysates (95). Thus, our data suggest that lipid aldehyde defence mechanisms are impaired in the diabetic retina and that this may represent a major mechanism through which toxic aldehydes and ALEs accumulate in this tissue during DR.

In addition to those enzymes described above, GSH-dependent aldehyde-metabolizing enzymes have been shown to play an important role in the detoxification of lipid aldehydes. Glutathione S-transferases (GSTs) act as a major cellular defense against 4-hydroxyalkenals and lipid hydroperoxides formed during lipid peroxidation reactions (192). The glyoxalase system also belongs to the group of GSH-dependent aldehyde metabolizing enzymes. This system, which is composed of two distinct metalloenzymes, glyoxalase I and glyoxalase II, specifically catalyzes the detoxification of oxoaldehydes such as GO and methylglyoxal (MGO) (179). Importantly, transgenic rats overexpressing glyoxalase I were shown to be protected against Müller cell dysfunction, pro-inflammatory signaling and capillary dropout in the retina during diabetes (193). These findings further reinforce the view that lipid peroxidation products and ALEs play a crucial role in development of DR.

## Therapeutic Approaches to Inhibit Lipoxidation Reactions in DR

The study of interventional strategies to prevent lipoxidation reactions and ALE accumulation during DR remains in its infancy. Several approaches are currently available to prevent the damaging effects of lipid aldehydes and ALEs in biological systems, including the use of antioxidants, direct aldehyde scavenging agents and the augmentation of endogenous aldehyde detoxification systems. Each of these approaches is described below, with particular emphasis placed on their potential for preventing lipid peroxidation and lipoxidation reactions during DR.

### Antioxidants

Antioxidants are enzymatic or non-enzymatic agents that can prevent undesired oxidation through their reaction with ROS or oxidation intermediates (98). As highlighted earlier, enzymatic antioxidants include SOD, catalase, glutathione peroxidase and glutathione reductase (59). Non-enzymatic antioxidants include vitamins E and C, β-carotene, GSH and flavonoids (60, 194).

Although GSH is one of the main cellular scavengers of ROS, to our knowledge there have been no studies directly assessing the efficacy of GSH supplementation for the treatment of DR. This is perhaps not surprising, given that oral GSH is rapidly hydrolyzed in the intestines into its constituent amino acids by the enzyme γ-glutamyl transpeptidase (195). Moreover, most cells are unable to absorb intact GSH (196). Nonetheless, GSH levels can be augmented by administration of GSH precursors such as cysteine and glycine (197). N-acetylcysteine (NAC) is a cysteine precursor for GSH synthesis that can also act as a thiol scavenger of ROS and lipid peroxidation products (198, 199). In diabetic rats, NAC treatment has been shown to protect against the development of retinal vasodegenerative pathology by ameliorating oxidative stress, the formation of 4-HNE protein adducts, VEGF production and inflammation (142, 200, 201). Similarly, glycine supplementation attenuates retinal vascular damage in STZ−diabetic rats and maintains retinal neuronal morphology and survival (202, 203). Lipoic acid is another endogenous thiol antioxidant that has been studied widely for its ability to inhibit the development of experimental DR. Treatment with lipoic acid suppresses the early upregulation of vasopermeability and angiogenic factors in the diabetic retina and prevents retinal leukostasis, an inflammatory feature of DR that contributes to vascular endothelial cell damage and capillary dropout (204–206). Administration of lipoic acid has also been reported to inhibit neurophysiological dysfunction and preserve retinal vascular and neuronal health during experimental DR (207–211). Despite these promising findings, however, in randomized control trials, daily administration of lipoic acid failed to prevent the occurrence of clinically significant DME in patients with T2D (212).

In addition to those discussed above, several other antioxidant agents have been tested in experimental animal models for their ability to prevent the vascular and neuronal abnormalities associated with DR (63). Trolox, a water soluble analog of vitamin E, was shown to partially prevent retinal capillary pericyte loss in diabetic rats by reducing membrane lipid peroxidation (213). It has also been reported that a combination of vitamin E and C suppressed $O_{2}^{\cdot -}$ production and led to a reduction in the formation of pericyte ghosts and acellular capillaries in the diabetic rat retina (74). Edaravone (3-methyl-1-phenyl-2-pyrazolin-5-one), an antioxidant that is used clinically to treat patients with ischemic brain injury and amyotrophic lateral sclerosis (ALS), was found to exert a neuroprotective role in retinas of diabetic mice, specifically preventing the upregulation of ROS and retinal ganglion cell loss (214). Given the large number of reports indicating that antioxidants may be effective in preventing the development of experimental DR, there remains a clear rationale for further studies evaluating their potential within a clinical setting.

### Scavenging Agents

Another approach for preventing the excessive accumulation of reactive aldehydes and ALEs is through the administration of direct lipid aldehyde scavenging agents. Several compounds have been identified that possess aldehyde sequestering properties, resulting in the formation of stable, non-toxic products (111). The relative reactivities of scavenging agents and thus their efficacies depend on the nucleophilic chemical functional group of the scavenger molecule along with the structure of the target aldehyde (215). In general, thiol and imidazole groups are superior to react with α, β-unsaturated aldehydes (e.g. ACR, 4-HNE, 4-HHE, etc.) (215, 216), whereas primary amines are more reactive with γ-ketoaldehydes (e.g. LGD2 and LGE2) (177). For further information on relative reactivities of scavenging agents *in vivo*, we direct readers to the following review (217). This section discusses the aldehyde-sequestering molecules that have been postulated or tested as potential therapeutics for the treatment of DR. A list of these agents and their chemical structures is contained in **Table 4**.

**Table 4:** Lipid aldehyde scavengers.

Name {abbreviation} <formula>	Structure
aminoguanidine {AG} < CH_7_ ${\text{N}}_{4}^{+}$ >	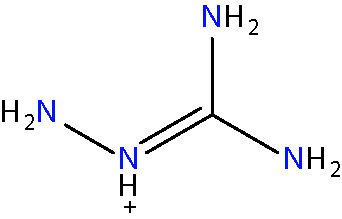
pyridoxamine {PM} < C_8_H_12_N_2_O_2_ >	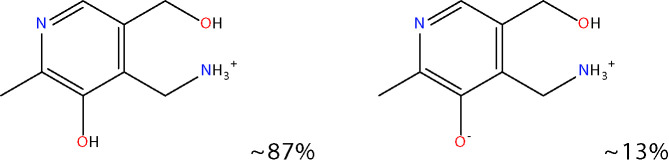
β-alanyl-L-histidine {carnosine} < C_9_H_14_N_4_O_3_ >	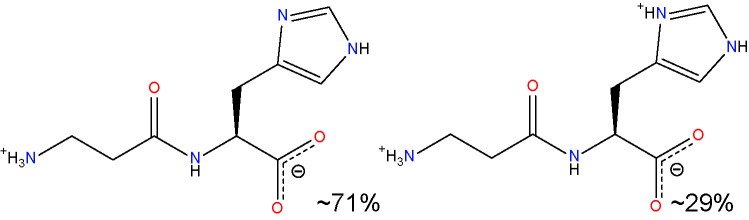
2-mercaptoethanesulphonate {MESNA} < C_2_H_5_O_3_ ${\text{S}}_{2}^{-}$>	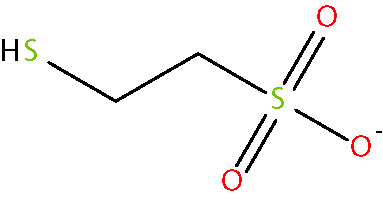
hydralazine {HDZ} < C_8_H_9_ ${\text{N}}_{4}^{+}$ >	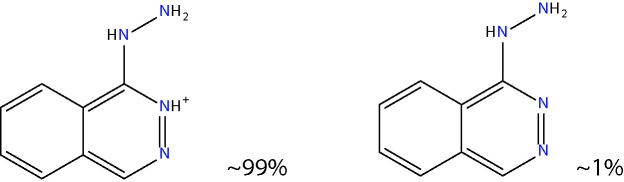
2-hydrazino-4,6-dimethylpyrimidine {2-HDP} < C_6_H_11_ ${\text{N}}_{4}^{+}$ >	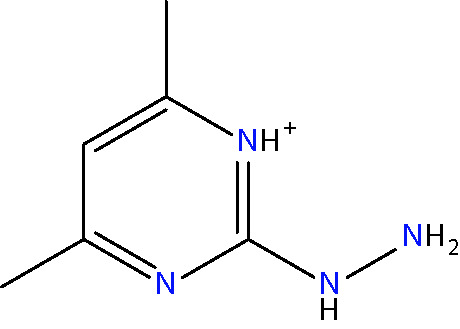

Chemicalize was used for prediction of microspecies abundance at pH 7, Dec, 2020, https://chemicalize.com/ developed by ChemAxon (http://www.chemaxon.com).

#### Aminoguanidine

Aminoguanidine (AG) is a polyreactive molecule, possessing both guanidine and hydrazine groups which endows it with strong nucleophilicity, making the molecule an effective scavenger of α,β-unsaturated aldehydes (218). AG has been shown to sequester both 4-HNE and MDA *in vitro* (98). Its ability to prevent AGE and ALE formation was associated with the inhibition of various diabetes-associated vascular complications such as nephropathy and diabetes-accelerated atherosclerosis (218). The treatment of diabetic rats with AG led to the prevention of retinal pericyte dropout and acellular capillary formation (219). In the same *in vivo* model, AG was found to decrease serum lipid peroxidation reactions, suggesting that its effects on DR development could, at least in part, be due to its ability to directly sequester lipid aldehydes (220). However, it is worth noting that AG also has a number of other mechanisms of action that could contribute to its ability to prevent the accumulation of lipid aldehydes and ALEs. For instance, AG has been shown to inhibit inducible nitric oxide synthase (iNOS), a molecule implicated in ROS production and the progression of vascular pathology in experimental diabetes (221). It is also capable of inhibiting SMOX, and as such, would be expected to reduce ACR production through blockade of the polyamine pathway (130). Clinical trials investigating the use AG in diabetic nephropathy were terminated early due to various side-effects, some stemming from the depletion of endogenous vitamin B6 derivatives (222). Interestingly, the first-line treatment for T2D metformin, another guanidine-like compound, has also been shown to have aldehyde sequestering abilities (223). Whether these effects, in addition to its glucose-lowering actions, contribute towards its ability to slow the development of DR and other diabetic complications remains to be established.

#### Pyridoxamine

Pyridoxamine (PM) is a naturally occurring isomer of vitamin B6 and is best known for its ability to block the Maillard reaction, and thus the formation of AGEs (224). However, PM is also known to directly scavenge lipid peroxidation-derived aldehydes (225). In diabetic rats, we have previously shown that administration of PM prevents the accumulation of GO- and ACR-derived ALEs, which was concomitant with a reduction in retinal vascular and neuroglial pathology (147, 172). Although PM appears promising as an early-stage therapeutic for DR, it has yet to be tested clinically within this setting. Clinical trials evaluating PM administration for the treatment of diabetic nephropathy, however, have been conducted, but these concluded that it lacked significant benefit (226). Safety concerns have also been raised regarding the use of this drug. Studies have shown that it may interfere with the activity and function of vitamin B6-dependent enzymes and metalloproteins due to its ion chelating activity (227). Aligning with these concerns, in diabetic rats, we found that PM appeared to negatively affect the neurophysiological function of retina, as determined by electroretinographic recording (47).

#### Carnosine

Carnosine (β-alanyl-L-histidine) is an endogenous, His-containing dipeptide, which is capable of sequestering aldehydes, such as 4-HNE, ACR and MDA (228, 229). Oral administration of carnosine has been tested pre-clinically in diabetic rats where it was found to reduce retinal vascular damage and capillary degeneration (230). This effect, however, was attributed to the induction of heat shock protein 27 (Hsp27) in retinal glial cells and the normalization of angiopoietin 2 (Ang2) signaling rather than being linked to its ability to scavenge aldehydes. Furthermore, significant increases in photoreceptor neurodegeneration were observed, raising concerns about the safety of this molecule in the treatment of DR. Another study in diabetic rats has suggested that carnosine may protect against microvascular changes in DR by suppressing mitogen-activated protein kinase (MAPK)/extracellular-signal-regulated kinase (ERK) signaling pathway (231). Recent investigations have demonstrated that a novel carnosine derivative, carnosinol, mitigates metabolic disorders of obesity by reducing carbonyl stress (232). This drug exhibited a good oral bioavailability and safety profile and would therefore be of interest to test within the context of DR.

#### 2-Mercaptoethanesulphonate

2-mercaptoethanesulphonate (MESNA) is a thiol-based scavenger, which sequesters aldehydes at rates faster than those of endogenous thiols such as glutathione (233). MESNA has been approved clinically as an adjunctive therapy for use with alkylating chemotherapy agents for nearly three decades (135). Although effective as a treatment for acute aldehyde toxicity associated with the use of these agents, the hydrophilicity of MESNA prevents it from readily entering cells, limiting its usefulness for the treatment of DR (47).

#### Hydrazino Compounds

Hydralazine (HDZ) and its analogous hydrazinophtalazines have been studied extensively for their ability to scavenge reactive aldehydes (111, 234). HDZ has been approved clinically as an anti-hypertensive agent for many years, although its use nowadays is mainly restricted to the treatment of uncontrollable hypertension in pregnancy (235). HDZ is a particularly potent ACR scavenger, being considerably more efficacious than AG, CAR and PM (229). Moreover, not only can this compound sequester free ACR, but it is also capable of binding to and neutralizing FDP-Lys adducts (236). The ACR sequestering ability of HDZ has been studied in a diabetic rat model of painful diabetic neuropathy. Daily intraperitoneal injections of HDZ reduced ACR-adduct formation in the spinal cord and alleviated diabetes-induced allodynia, hyperalgesia, and microglial activation (237).

The potential clinical application of HDZ as a therapeutic for DR is complicated by its cardiovascular actions. However, since the hydrazine groups on HDZ are known to be responsible for its aldehyde-scavenging abilities (234), but not its anti-hypertensive effects (238), it is apparent that other compounds containing hydrazino groups could have therapeutic value for DR. With this in mind, our group recently screened a variety of hydrazino compounds using an ELISA based method, identifying 2-hydrazino-4,6-dimethylpyrimidine (2-HDP) as a highly effective scavenger of ACR with no obvious cardiovascular effects (102). Oral administration of this compound in the drinking water of diabetic rats prevented retinal FDP-Lys accumulation, Müller cell gliosis, oxidative stress, pro-inflammatory signaling and microglial activation. Furthermore, this drug improved the neurophysiological function of the retina. Ongoing studies in our laboratory are examining whether this drug is also capable of preventing the neuro- and vasodegenerative complications associated with DR.

### Induction or Augmentation of Endogenous Aldehyde Detoxification Systems

In addition to the use of scavenging agents, another approach to prevent retinal accumulation of lipid peroxidation and lipoxidation products is through the augmentation of endogenous defense mechanisms. Presently, there are few drugs that are clinically useful for increasing the activity and expression of aldehyde metabolizing enzymes. Perhaps the most promising to date, however, is ALDA-1, which was originally developed as a small molecule activator of ALDH2, but more recently has been shown to also activate ALDH1a1 with similar potency (239, 240). This drug has generated some encouraging results in early-stage pre-clinical DR studies. Specifically, ALDA-1 was shown to be effective in enhancing SOD activity and decreasing the expression of pro-inflammatory and angiogenic mediators in the retinas of diabetic rats of 1-mo disease duration (241). Nonetheless, it seems likely that the efficacy of this drug may be reduced as diabetes progresses due to the observed downregulation of ALDH enzymes (see *Endogenous Detoxification of Lipid Aldehydes and DR* above).

Many of the aldehyde metabolizing enzymes known to be present in retina are inducible by their substrates and pharmacological agents (240, 242). Various studies have shown that constitutive androstane receptor (CAR) activators and pregnane X receptor (PXR) ligands induce strong upregulation of ALDH and AKR gene expression in tissues of experimental rodent models (240, 242). These receptor systems could represent new and attractive targets to inhibit lipoxidation reactions in the context of DR and other diabetic complications.

Recent developments in the use of AAV vectors for the efficient delivery of genes of interest to retinal neurons and macroglial cells *in vivo* (243), suggests that the use of gene therapy approaches may also provide an effective means of enhancing ALDH and AKR expression and activity in the diabetic retina. An advantage of gene therapy is that it would facilitate sustained local expression of aldehyde detoxifying enzymes, reducing the likelihood of systemic side effects and the need for regular intravitreal injections. To our knowledge there have been no studies to date specifically testing gene therapy approaches to target lipoxidation reactions in DR, but this constitutes a promising strategy that is worthy of future investigation.

## Conclusions

Given a combination of high PUFA content, high oxygen consumption, and a high level of metabolic activity, the retina is a site of high ROS, LOOH, and lipid aldehyde production. The accumulation of damaging levels of lipid aldehydes and ALEs in DR is a consequence of a shift in the balance between the rate of production and clearance. As an increasing body of evidence suggests this accumulation is tied to the pathogenesis of DR, lipid peroxidation and lipoxidation have emerged as important therapeutic targets for the treatment of this condition. Two of the most promising approaches are the use of aldehyde scavenging molecules or gene therapeutic enhancement of clearance and detoxification pathways. These approaches, however, require further developments. We need new, potent, and safe scavengers that target lipid aldehydes in the eye. Gene therapy will require identification of the cell types and clearance pathways that could be best exploited to enable an efficacious approach. While total lipid composition, reactive aldehyde generation, and ALE accumulation change in DR, we do not yet understand if these changes correlate with the focal pathology that typically develops during this disease (14). Powerful lipidomic mapping methods, like high resolution MALDI mass spectrometry (244, 245) could reveal such correlations. There is also currently a limited understanding of whether and how different reactive aldehydes interact with one another and their relative contributions to the development of DR pathology. Additionally, an important consideration in the development of therapeutic strategies will be to confirm that they do not cause detrimental effects by inhibiting physiological cell signaling by low levels of endogenous aldehydes. Also, revealing scavenger molecule pharmacokinetics (246–249) and their precise biochemical interactions with diverse aldehyde species (119, 215, 250) whilst minimizing off-target effects (111) will be crucial in the advancement of these therapeutics. Future progress in these areas offers the exciting opportunity to prevent and potentially reverse lipid aldehyde and ALE accumulation in DR.

## Author Contributions

JA, CM, and TF prepared the initial draft of the manuscript. TC, PB, ET, TF, JA, and AS edited the manuscript for intellectual content. Figures and Tables were created by ET, JA, TC, and CM. All authors contributed to the article and approved the submitted version.

## Funding

JA was supported by a research grant from Diabetes UK (18/0005791). PB and TF were supported by research grants from Health & Social Care R&D Division, Northern Ireland (STL/4748/13) and the Medical Research Council (MC_PC_15026).

## Conflict of Interest

The authors declare that the research was conducted in the absence of any commercial or financial relationships that could be construed as a potential conflict of interest.
